# Factors Influencing the Effectiveness of Botulinum Toxin Therapy in Bruxism Management

**DOI:** 10.3390/toxins17080384

**Published:** 2025-07-31

**Authors:** Azusa Furuhata, Kazuya Yoshida, Shiroh Isono

**Affiliations:** 1 Department of Internal Medicine, Nippon Dental University, Tokyo 102-8158, Japan; 2 Furuhata Snoring and Sleep Disorder Research Institute and Furuhata Dental Clinic, Tokyo 107-0052, Japan; 3 Department of Oral and Maxillofacial Surgery, National Hospital Organization, Kyoto Medical Center, Kyoto 612-8555, Japan; omdystonia@gmail.com; 4 Department of Anesthesiology, Chiba University, Chiba 260-8677, Japan; shirohisono@yahoo.co.jp

**Keywords:** botulinum neurotoxin, botulinum toxin therapy, bruxism, bite force, masseter muscle, electromyography, aging, muscle pain, sex difference, sleep quality

## Abstract

A total of 304 patients with bruxism (206 women, 98 men; mean age: 52.5 years) received 25 units of botulinum toxin injected into the bilateral masseter muscles; the changes in various clinical symptoms and their contributing factors were analyzed 2 months after treatment. The mean masseter muscle electromyographic amplitude (189 μV) and maximal bite force (618.4 N) significantly decreased after botulinum toxin therapy compared to that at baseline (55.4 μV, 527.3 N, respectively; *p* < 0.001). Maximal mouth opening (44 mm), sleep quality (visual analog scale: 5.3), shoulder and neck stiffness (6.7), and headache (5.4) significantly improved after the injection (47.3 mm, 6.6, 4.7, and 2.6, respectively; *p* < 0.001). Multivariate analysis revealed that the mean masseter electromyographic amplitude reduction rate was significantly affected by age, sex, and baseline amplitude (all *p* < 0.001); the maximal bite force reduction rate was influenced by age (*p* < 0.001), sex (*p* = 0.007), and baseline bite force (*p* = 0.008). Age, sex, and muscle activity may affect the therapeutic effects. A more effective outcome for bruxism can be achieved using a tailored approach involving dose adjustment, thereby preventing the side effects attributed to excessive dosage.

## 1. Introduction

Bruxism is classified as both sleep and awake bruxism, which are defined as masticatory muscle activities occurring during sleep and wakefulness, respectively [1]. Sleep bruxism is characterized as rhythmic or nonrhythmic, while awake bruxism is characterized by repetitive or sustained tooth contact and/or bracing or thrusting of the mandible [1].

Bruxism is reportedly associated with tooth wear or destruction; failure of dental prostheses or implants; pain in the teeth, masticatory muscles, or temporomandibular joints (TMJs); temporomandibular disorders; masseter muscle hypertrophy; or tension headache [1,2]. Bruxism is a major problem in dental clinical practice and has long attracted the attention of several dental clinicians and researchers. Although the etiology of bruxism has not yet been fully elucidated, a multifactorial etiology was postulated, including biological, psychological, and exogenous causes [1,2,3,4]. A prevalence of 5.0% was noted for awake bruxism and 16.5% for sleep bruxism [5]. An umbrella review reported that the prevalence of awake bruxism was 22–30%, that of sleep bruxism was 1–15%, and that among children and adolescents was 3–49% [2].

Treatment modalities include the oral administration of muscle relaxants and analgesics, splint therapy, and physical therapy. However, these treatments are considered insufficient for many patients. In recent years, botulinum neurotoxin (BoNT) injection into the masticatory muscles has been considered a promising alternative [6]. Several randomized controlled trials have demonstrated its therapeutic effectiveness [7,8,9,10,11,12,13,14,15,16,17,18]. However, owing to the variability in study designs and the absence of standardized treatment protocols, drawing uniform conclusions is challenging. BoNT therapy, which is frequently used in clinical dentistry, is considered an effective treatment. Patients with bruxism who complain of various symptoms often experience improvements in headaches, stiff neck and shoulders, and sleep quality associated with bruxism following BoNT therapy, in addition to reduced muscle pain and discomfort. This study aimed to investigate the factors influencing these therapeutic effects.

## 2. Results

A total of 25 units of BoNT-A (Botulax^®^, Hugel Inc., Seoul, Republic of Korea) was injected into the masseter muscles bilaterally in 304 patients with bruxism (206 women, 98 men, mean age: 52.5 ± 15.9, 18–89 years). No obvious complications were observed following BoNT therapy.

### 2.1. Changes in the Main Measurement Items After Treatment

**Figure 1:**
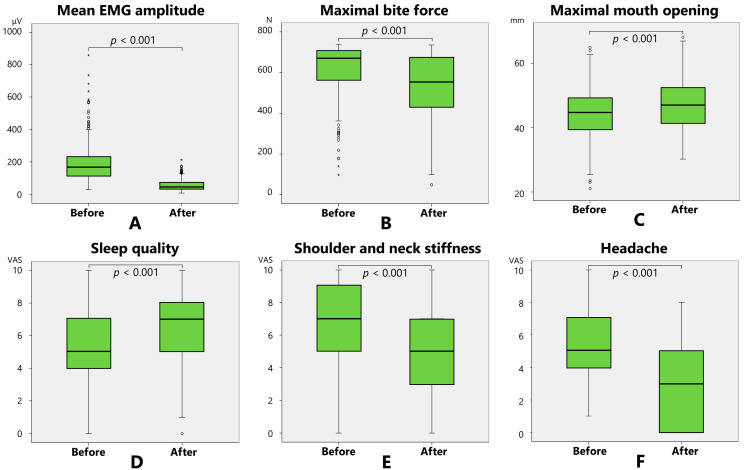
Mean masseter muscle electromyographic (EMG) amplitude (**A**), maximal bite force (**B**), maximal mouth opening (**C**), sleep quality (**D**), shoulder and neck stiffness (**E**), and headache (**F**) improved significantly after botulinum neurotoxin (BoNT) therapy compared to baseline.

The masseter muscle electromyographic (EMG) amplitude and maximal bite force were significantly (*p* < 0.001, Wilcoxon’s signed-rank sum test) decreased 2 months after BoNT therapy compared to those at baseline (Table 1, Figure 1). Maximal mouth opening, sleep quality, shoulder and neck stiffness, and headache were significantly (*p* < 0.001) alleviated following the BoNT injections (Table 1, Figure 1).

### 2.2. Factors Affecting Symptoms and Effects

Among these parameters, three variables (age, sex, and baseline EMG amplitude) for the EMG reduction rate and three parameters (age, sex, and baseline bite force) for the maximal bite force reduction rate were selected as predictors after stepwise analysis (Table 2).

Multivariate analysis demonstrated that the mean EMG amplitude reduction rate was significantly affected by the age (*p* = 0.001), sex (*p* < 0.001), and baseline amplitude (*p* < 0.001), and the maximal bite force reduction rate was influenced by the age (*p* < 0.001), sex (*p* = 0.007), and baseline bite force (*p* = 0.008) (Table 2).

#### 2.2.1. Mean EMG Amplitude

**Figure 2:**
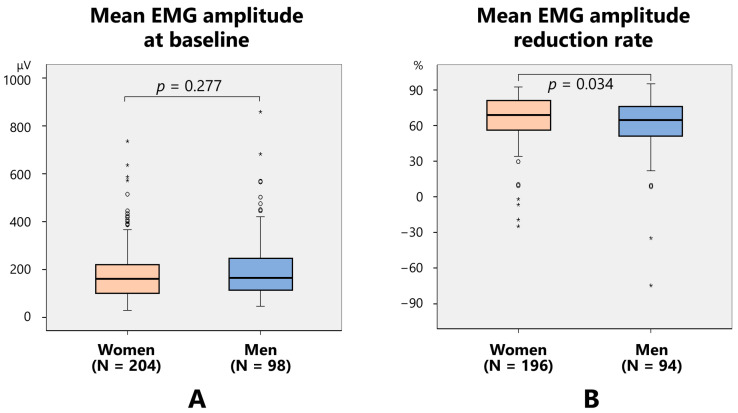
Comparison between women and men regarding the mean electromyographic (EMG) amplitude at baseline (**A**) and the mean EMG amplitude reduction rate (**B**).

Mean EMG amplitude at baseline did not vary between women (183.1 ± 109.8 μV) and men (205.8 ± 139.2 μV) (Figure 2A). The EMG amplitude following treatment was significantly (*p* < 0.001, Mann–Whitney’s U test) lower in women (50.4 ± 28.7 μV) than in men (65.6 ± 38.3 μV). The rate of EMG amplitude reduction was significantly higher in women (66.9 ± 18.4%) than in men (61.1 ± 24.7%) (*p* = 0.034) (Figure 2B).

**Figure 3:**
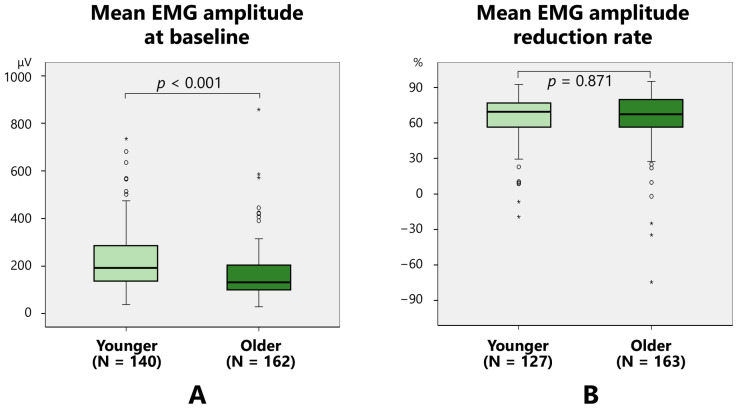
Comparison between younger (<52.5 years old) and older (>52.5 years old) participants considering the mean electromyographic (EMG) amplitude at baseline (**A**) and the mean EMG amplitude reduction rate (**B**).

The baseline mean EMG amplitude was significantly higher in younger (<52.5 years old; mean age of the total participants) (223.6 ± 128.3 μV) groups than older (over 52.5 years old) participants (161.8 ± 105.3 μV) (*p* < 0.001) (Figure 3A). The reduction rate of the mean EMG amplitude did not vary between younger (64.8 ± 19.3%) and older (65.2 ± 22.0%) groups (Figure 3B).

#### 2.2.2. Maximal Bite Force

**Figure 4:**
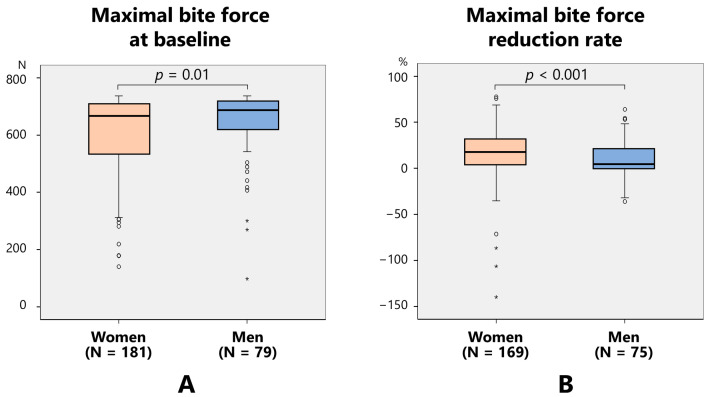
Comparison of the maximal bite force between women and men at baseline (**A**) and reduction rate (**B**).

Maximal bite force at baseline was significantly lower in women (605.9 ± 133.6 N) than in men (646.2 ± 114.4 N) (*p* = 0.01) (Figure 4A). The maximal bite force after treatment was significantly lower in women (507.7 ± 162.3 N) than in men (580.4 ± 145.5 N (*p* < 0.001)). The reduction rate of the maximal bite force was significantly higher in women (16.5 ± 26.6%) than in men (10.1 ± 18.3%) (Figure 4B).

**Figure 5:**
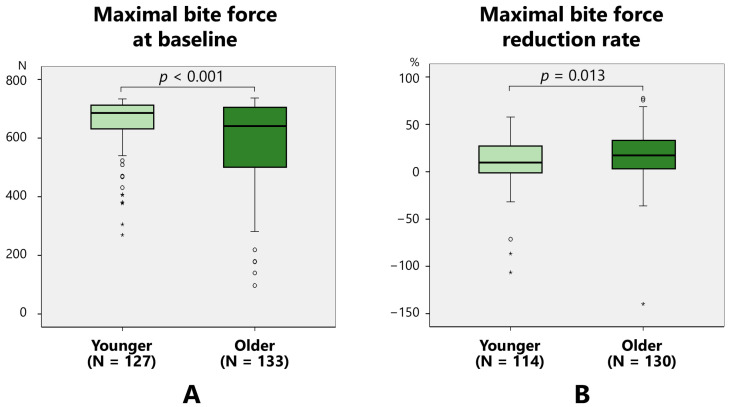
Comparison of the maximal bite force between younger and older groups at baseline (**A**) and reduction rate (**B**).

The baseline maximal bite force was significantly higher in the younger group (652.4 ± 93.3 N) than in the older group (585.4 ± 149.0 N) (*p* < 0.001) (Figure 5A). The reduction rate of the maximal bite force was significantly higher in older (18.1 ± 25.3%) patients than in younger (10.4 ± 22.9%) (*p* = 0.013) patients (Figure 5B).

**Figure 6:**
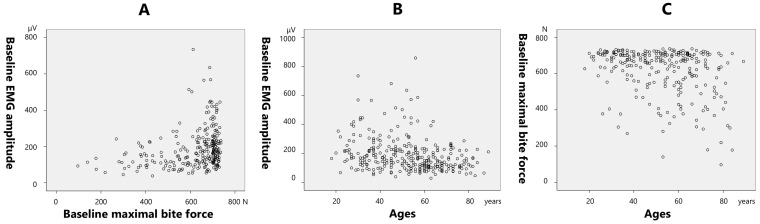
Correlation between the baseline electromyographic (EMG) amplitude and maximal bite force (**A**), and correlations between ages and baseline EMG amplitude (**B**) and maximal bite force (**C**).

The maximal bite force before treatment demonstrated a significantly positive correlation (0.284; *p* < 0.001) with the pretreatment EMG amplitude (Figure 6A). Pretreatment EMG amplitude demonstrated a significantly negative correlation with age (−0.266; *p* < 0.001) (Figure 6B). Pretreatment bite force demonstrated a significantly negative correlation with age (−0.315; *p* < 0.001) (Figure 6C).

**Figure 7:**
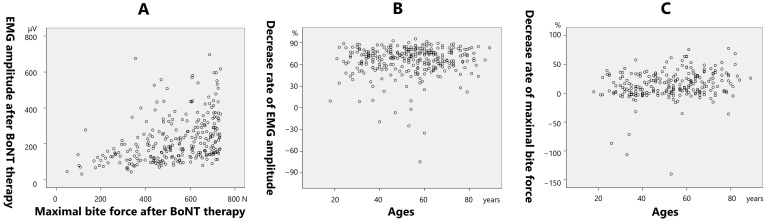
Correlation between the post-treatment bite force and post-treatment electromyographic (EMG) amplitude (**A**), correlation between post-treatment EMG amplitude reduction rate and age (**B**), and correlation between the maximal bite force reduction rate and age (**C**). Note that several patients demonstrated increased muscle activity and bite force following botulinum neurotoxin (BoNT) therapy (**B**,**C**).

The post-treatment bite force demonstrated a significantly positive correlation with the post-treatment EMG amplitude (0.403; *p* < 0.001) (Figure 7A). The rate of decrease in the EMG amplitude after treatment demonstrated a significantly positive correlation with age (0.346; *p* < 0.001) (Figure 7B). The rate of decrease in the maximal bite force demonstrated a significantly positive correlation with age (0.223; *p* < 0.001) (Figure 7C).

#### 2.2.3. Maximal Mouth Opening

**Figure 8:**
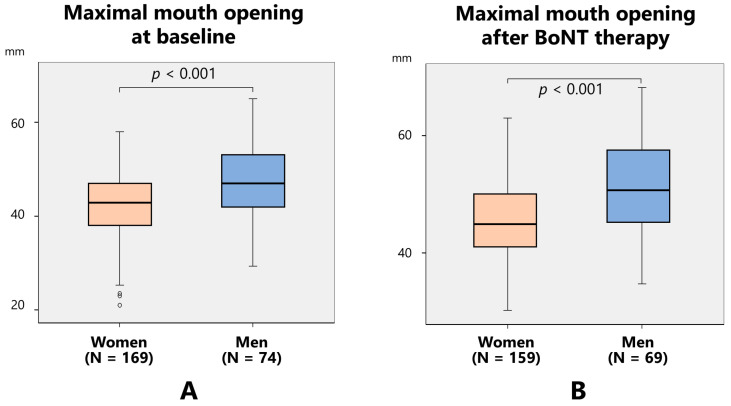
Maximal mouth opening at baseline (**A**) and after botulinum neurotoxin (BoNT) therapy (**B**). Maximal mouth opening was significantly larger in men than in women.

Maximal mouth opening at baseline was significantly larger in men (47.7 ± 7.8 mm) than in women (42.5 ± 7.3 mm) (*p* < 0.001) (Figure 8A), and that after BoNT therapy was significantly larger in men (51.3 ± 7.9 mm) than in women (45.7 ± 6.8 mm) (*p* < 0.001) (Figure 8B).

**Figure 9:**
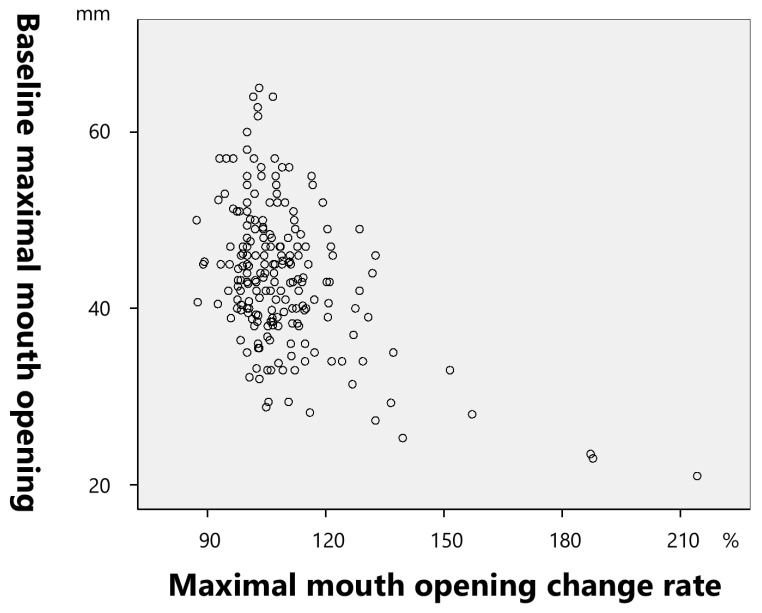
Correlation between the rate of change in the maximal mouth opening and baseline maximal mouth opening.

The rate of change in the maximal mouth opening demonstrated a significantly negative correlation with the pretreatment maximal mouth opening (−0.461; (*p* < 0.001) (Figure 9).

#### 2.2.4. Sleep Quality

Sleep quality at baseline demonstrated a significantly positive correlation with age (0.252; *p* < 0.001), negative correlation with the EMG amplitude before treatment (−0.131; *p* = 0.05), and positive correlation with the EMG reduction rate (0.131; *p* = 0.04). The sleep quality change rate demonstrated a significantly positive correlation with the EMG amplitude before treatment (0.171; *p* = 0.008) and negative correlation with the sleep quality before treatment (−0.607, *p* < 0.001).

#### 2.2.5. Shoulder and Neck Stiffness

**Figure 10:**
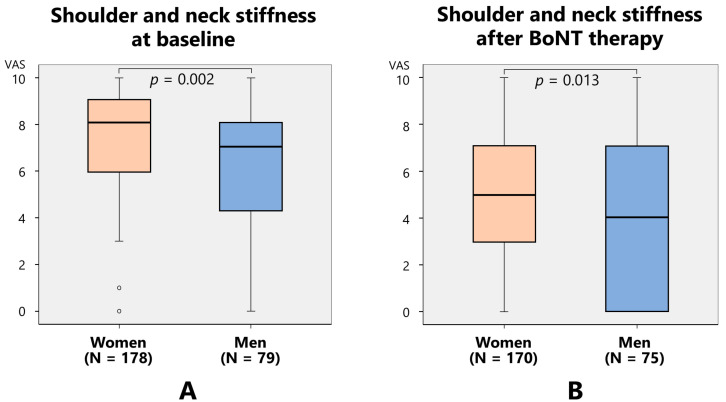
Before treatment, shoulder and neck stiffness was significantly higher in women than in men (**A**), and after treatment, neck and shoulder stiffness was significantly higher in women than in men (**B**).

At baseline, neck and shoulder stiffness were significantly higher in women (7.1) than in men (5.8) (*p* = 0.002). After treatment, neck and shoulder stiffness were significantly higher in women (5.0) than in men (4.0) (*p* = 0.013) (Figure 10).

Shoulder and neck stiffness at baseline demonstrated a significantly negative correlation with age (−0.185; *p* = 0.003), sleep quality before treatment (−0.22; *p* = 0.001), and sleep quality after treatment (−0.159; *p* = 0.016). Stiffness after treatment demonstrated a significantly negative correlation with sleep quality before (−0.165; *p* = 0.012) and after treatment (−0.204; *p* = 0.002). The stiffness reduction rate exhibited a significant correlation with the stiffness before treatment (0.297; *p* < 0.001).

#### 2.2.6. Headaches

Pretreatment headaches were significantly correlated with pretreatment stiffness (0.319; *p* < 0.001) and demonstrated a significantly (*p* = 0.015) negative correlation (−0.189) with age. The headache reduction rate was significantly positively correlated with the stiffness reduction rate (0.238; *p* = 0.003).

## 3. Discussion

This study is the first to report that age, sex, and muscle strength have significant effects on the treatment outcomes of BoNT therapy for bruxism, thereby suggesting the importance of a tailored approach. More effective treatment and side effects associated with excessive dosages can be avoided by adjusting the optimal dosage for each patient.

### 3.1. Diagnosis of Bruxism

The diagnosis of bruxism in previous studies was based on a combination of both subjective and objective criteria, including the evaluation of clinical signs and symptoms, such as muscle pain, grinding, attrition of the posterior teeth, questionnaires, and electromyography. A grading system for bruxism was proposed to determine the likelihood of an assessment of bruxism actually yielding a valid outcome: (1) possible sleep/awake bruxism, based only on a positive self-report; (2) probable sleep/awake bruxism, based on a positive clinical inspection, with or without a positive self-report; and (3) definite sleep/awake bruxism, based on a positive instrumental assessment, with or without a positive self-report and/or positive clinical inspection [1]. The most recent systematic review of BoNT therapy for bruxism [17] evaluated 12 randomized controlled trials [5,6,7,8,9,10,11,12,13,14,15,16]. Patients were diagnosed with definite bruxism in nine studies [5,7,8,9,10,12,13,14,16]. Two studies [10,14] applied the International Classification of Sleep Disorders, whereas one study followed the international consensus on the definition and diagnosis of bruxism [16]. In this study, bruxism was comprehensively diagnosed based on the patients’ partners’ comments, examination results by referring doctors, and many other clinical symptoms. Recently, polysomnography with audio–video recordings or ambulatory EMG devices has been used for definite diagnoses [4]. Sleep bruxism and sleep quality should be assessed objectively using a validated polysomnography method. Palpation, EMG, and occlusal force measurements are helpful in identifying the target muscles [19]. In most cases, the masseter muscle is injected first. Additionally, the temporalis muscle is injected. In cases of medial pterygoid muscle tenderness, the effect diminishes upon continuous injections into the masseter and temporalis muscles. The medial pterygoid muscle is also injected [20]. In cases of severe grinding, the lateral pterygoid muscles are often sensitive [20]. The occlusal force must be measured before and after each BoNT injection because long-term repeated injections can result in occlusal force reduction and masticatory disturbance [19].

Oromandibular dystonia is a focal dystonia characterized by contractures of the masticatory and/or lingual muscles [19]. Patients with oromandibular dystonia first consult dentists or oral surgeons and are likely to be diagnosed with bruxism or temporomandibular disorders by dental professionals [21]. Many patients diagnosed with awake bruxism exhibit jaw closing oromandibular dystonia [21]. The effect of treatment of bruxism in patients with jaw closing dystonia is limited, and differential diagnosis between awake bruxism and jaw closing dystonia is essential. Notably, many dental professionals are interested in studying bruxism but not OMD. Therefore, raising awareness of OMD among dentists and oral surgeons who are likely to encounter patients with OMD first is critical [21].

The clinical application of BoNT therapy has increased recently. Most patients with bruxism experience symptomatic relief through traditional methods such as oral medications and splints. Therefore, BoNT therapy should only be considered in severe cases when other treatments are deemed ineffective [19]. Prolonged and extremely severe bruxism can cause the development of excessive tendonous tissue at the anterior margin of the masseter muscle, resulting in masticatory muscle tendon–aponeurosis hyperplasia and severe trismus [22]. BoNT therapy is ineffective in such cases, warranting coronoidotomy [22].

### 3.2. Methodology of BoNT Therapy for Bruxism

Methodology, including study design, BoNT dosage, injection sites and methods, follow-up duration, and participants, varied widely in randomized controlled trials [6]. The mean age of participants ranged from 25 [6,7] to 58 years [13]. Previous reports on the effectiveness of BoNT therapy for bruxism included participants younger than those included in this study. In dental clinical practice, a relatively large number of elderly patients with bruxism are encountered, with the average age in this study being 52.5 years.

The average number of participants in the 12 previous randomized controlled trials was 28. In the present study, the data were based on 304 patients, which is considered a sufficient number of participants.

The total dosage of BoNT-A injections administered to the masseter muscles ranged from 20 units [15] to 120 units [10]. In the temporalis muscles, the dosage ranged from 30 units [14] to 80 units [10]. Cruse et al. [14] injected 15 units into each medial pterygoid muscle. However, minimizing the number of muscles injected and reducing the BoNT dose are recommended.

The lack of an established treatment protocol has led to the development of various BoNT therapies. Most studies have evaluated the results of a single BoNT administration method without varying the muscles or injection points. Only one study [7] compared the application in the masseter muscles with and without the temporalis muscle.

In most of the studies, the follow-up period was short. Only one study reported that the effects were maintained for up to 1 year after treatment [8]. A longer follow-up duration is warranted to determine the long-term effectiveness of BoNT in treating bruxism.

This study analyzed real-world data from clinical dentistry to examine the effects of BoNT therapy on the bilateral masseter muscles in 304 patients with bruxism of various symptoms across a wide age range, from the clinical perspective of electromyography and maximal bite force, to determine the factors influencing the treatment outcomes. Because this was an observational study without a control group, the influence of the placebo effect could not be ruled out. In future, it will be necessary to conduct evidence-based research using a randomized controlled study design.

### 3.3. Muscle Pain

BoNT acts as a muscle relaxant and exerts analgesic effects in neuromuscular disorders. Therefore, BoNT injection into painful masticatory muscles is considered an ideal treatment modality for muscle pain associated with bruxism [23]. A significant reduction in the pain scores in the BoNT-A group compared with the placebo group was demonstrated in three studies [8,10,15], whereas one study [8] reported the same reduction in the experimental group compared with conventional treatments. In contrast, five other studies reported the efficacy of BoNT-A in addressing bruxism-related myofascial pain symptoms [5,8,12,15,16]. In addition, Kaya and Ataoglu reported that both occlusal splints and BoNT-A were effective in alleviating the pain associated with bruxism [12]. However, BoNT-A was found to be slightly less effective than occlusal splints in reducing pain. Notably, BoNT-A injections still provided a significant reduction in the pain symptoms, making them a viable alternative treatment option [12]. Two other studies [10,14] reported contradictory results in terms of pain reduction after BoNT-A injections. No evidence was found for a change in the pain intensity as measured by the short-form McGill Pain Questionnaire [14] or the visual analog scale (VAS) [10,14] compared to the control group and before the injection. Eight studies employed the VAS [5,8,9,10,12,14,15,16], whereas only one used the short-form McGill Pain Questionnaire [14]. In this study, the tenderness was measured using standardized methods and established equipment (Figure 13). Therefore, the test pressure, which can be kept constant for each patient before and after treatment, is highly reliable.

### 3.4. Muscle Activity and Bite Force

Three studies [7,9,15] indicated the efficacy of BoNT-A injections for sleep bruxism, as evaluated by the reduction in the intensity of both the masseter and temporalis muscles compared with the placebo control groups. However, this reduction was confirmed only in the masseter muscles in two other studies [6,11]. Three studies reported that the administration of BoNT-A into the masseter muscle [6,11] and both the temporalis and masseter muscles reduced muscle activity compared to that at baseline [14]. Cruse et al. demonstrated a decrease in the bruxism index of the masticatory muscles evaluated by EMG between the experimental and control groups [14]. The heterogeneity of the results may be attributed to methodological differences, such as injection methods, including doses, injection sites, points, and depth. The masseter muscle comprises superficial and deep parts, and BoNT acts on nerve endings; therefore, injecting it into the endplate zone is effective [23]. However, whether the authors of the abovementioned studies considered these factors is unclear.

**Figure 11:**
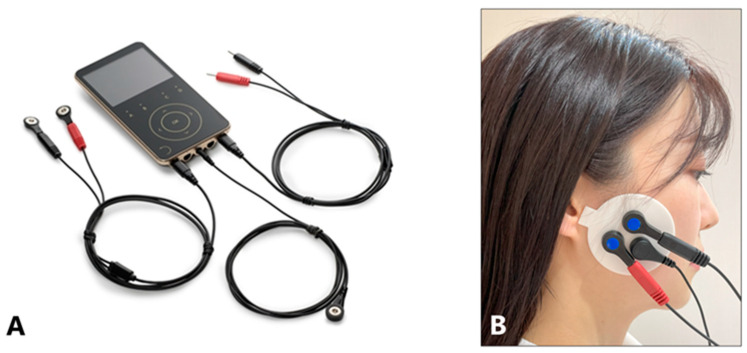
Myonyx (**A**), electromyographic (EMG) activity was recorded from bilateral masseter muscles (**B**).

In the present study, EMG and maximal bite force were assessed using standardized methods and established equipment (Figure 10 and Figure 11). The reproducibility of measurements before and after treatment must be high. The EMG amplitude of the masseter muscle decreased significantly from 189 μV to 55.4 μV, whereas the decrease in bite force was smaller, from 618.4 N to 527.3 N. The masseter contributes approximately 43% of the intrinsic strength of the jaw elevator muscles, 36% of the strength of the temporalis muscle, and 21% of the strength of the medial pterygoid muscle [24]. In this study, due to the diagnosis before treatment, BoNT injections were administered only to the bilateral masseter muscles. Therefore, the EMG activity of the masseter muscles decreased by an average of more than 70%, whereas the decrease in bite force was only approximately 15%. It is important to minimize the dose of BoNT therapy to reduce the cost borne by patients and the risk of long-term antibody production during the longitudinal treatment of bruxism. A decrease in the bite force can lead to masticatory disturbance, and the injected muscle should be selected after a thorough examination [19].

A reduction in muscle activity is one of the preferred effects of BoNT for bruxism. Multivariate analysis revealed that age, sex, muscle activity, and bite force could influence the efficacy of BoNT therapy for bruxism. There was no difference in the reduction rate of the EMG amplitude between the young and older groups (Figure 3B); however, a significant difference was observed in the reduction rate of the bite force between the groups (Figure 5B). Because it takes longer for the effects of BoNT to appear in older patients than in younger patients, it is speculated that the masseter and temporalis muscles were not fully functional at the time of the 2-month examination period in this study. In the present study, the maximal bite force at baseline demonstrated a significant positive correlation with the pretreatment EMG amplitude (Figure 6A). Age was significantly negatively correlated with the retreatment EMG amplitude (Figure 6B) and maximal pretreatment bite force (Figure 6C). A progressive decline in the skeletal musculature mass of the body is well known [25,26]. The masseter muscle area decreases with age [27], bite force also decreases with age, with a sex predilection [28,29]. The post-treatment maximal bite force demonstrated a significant positive correlation with the post-treatment EMG amplitude (Figure 7A). The rate of decrease in the EMG amplitude after treatment was significantly positively correlated with age (Figure 7B), and the rate of decrease in the maximal bite force was significantly positively correlated with age (Figure 7C). The maximal bite force after treatment was significantly lower in women than in men. Although the maximal bite force decreased significantly after BoNT therapy, there were several cases in which the maximal bite force increased after treatment (Figure 7C). This can be interpreted as follows: patients with muscle pain were unable to bite properly before treatment owing to pain during measurement of the maximal bite force; however, after treatment, muscle pain was alleviated, and they were able to exert sufficient bite force. Although setting a threshold of bite force change as an indicator of therapeutic effect for BoNT therapy into the jaw-closing muscles might be useful for clinicians, a common metric may not adequately reflect treatment efficacy across all patients. Thus, significant differences exist in the bite force and effects of BoNT therapy based on the age and sex. Therefore, a tailored approach is warranted to determine the minimum number of muscles that should be injected and the optimal dose for each patient, considering factors such as age, sex, and muscle strength. Although ultrasound guidance was not employed in the present study, we acknowledge its increasing role in enhancing both the safety and precision of BoNT therapy in clinical settings. Some researchers suggested the importance of ultrasound guidance of personalized BoNT therapy for cervical dystonia [30,31]. A recent detailed review on ultrasound-guided BoNT injection into the masseter muscle emphasized the advantages of ultrasound guidance in improving the precision of injection compared to blind methods [32]. Findings of EMG and bite force and ultrasound metrics could facilitate more personalized and accessible treatment strategies. Incorporating ultrasound into future research and routine practice may help establish more standardized, effective, and safer treatment protocols for bruxism and other oromandibular disorders.

### 3.5. Sleep Quality

In many patients, sleep quality is significantly affected by sleep bruxism. The Epworth Sleepiness Scale [10,14] and Pittsburgh Sleep Quality index [10,13] were used to evaluate this outcome. One study [13] highlighted that patients with bruxism experienced enhanced sleep quality following BoNT-A administration. These three trials indicated that the scale or index was alleviated following BoNT administration [10,13,14]. A previous study indicated that the total sleep time tended to improve [10].

In the present study, sleep quality before treatment demonstrated a significant positive correlation with age, negative correlation with EMG amplitude at baseline, and positive correlation with the EMG decrease rate. The findings indicate that older patients tended to exhibit higher sleep quality in this study. Patients with good sleep quality demonstrated low EMG amplitude, resulting in improvement in sleep quality. The average age of the study participants was 52.5 years old; participants in their 60s or older may have already retired, while those in their 40s and 50s may be subject to hard work and work-related stress that might affect their sleep quality. The sleep quality change rate demonstrated a significant positive correlation with the EMG amplitude before treatment and a significant negative correlation with sleep quality at baseline. These results suggest that patients with a lower sleep quality and higher EMG activity benefit more from BoNT therapy. However, the data in the present study were based on VAS scores, which are open to bias. Therefore, it is prudent to draw conclusions based on this study. Thus, further studies using more objective assessment tools are warranted. In this study, the maximal mouth opening improved significantly after BoNT injections (Table 1, Figure 1). A significant negative correlation was observed between the rate of change in the mouth opening and sleep quality before treatment. BoNT administration to the masseter muscle relieved hypertonia in the masseter muscle and increased the amount of maximal mouth opening. The improvement rate seemed higher in cases with severe restrictions on mouth opening and lower sleep quality.

### 3.6. Other Secondary Outcomes

Other secondary outcomes have rarely been analyzed in previous randomized controlled trials. In this study, shoulder or neck stiffness and headaches were carefully assessed and evaluated using the VAS.

Shoulder or neck stiffness at baseline was significantly negatively correlated with age and sleep quality before and after treatment. These results indicate that older patients tend to have less shoulder or neck stiffness; patients with severe stiffness have lower sleep quality even after treatment. Participants in their 60s or older may have retired, while younger participants may have demanding job profiles, which might have affected their shoulder and neck stiffness. Before and after treatment, the neck and shoulder stiffness was significantly higher in women than in men. Sex variations in the shoulder and neck stiffness may exist. Stiffness after treatment was significantly negatively correlated with sleep quality at baseline and after treatment. The stiffness reduction rate was significantly correlated with the stiffness before treatment. This may imply that sleep quality after treatment remained insufficient if the stiffness was not alleviated satisfactorily.

Pretreatment headaches were significantly correlated with pretreatment stiffness and demonstrated a significant negative correlation with age. The headache reduction rate was significantly and positively correlated with the stiffness reduction rate. The findings may show that baseline headaches are associated with stiff shoulders or necks; older patients tended to have fewer headaches, with the stiffness improving with the improvement in headaches. Differences may also exist in headaches between retired and younger people with demanding job profiles.

The findings of the present study offer valuable insights into the therapeutic potential of BoNTs in terms of secondary outcomes. The VAS was used for other evaluation items; however, as a placebo effect may exist, more objective evaluation methods are needed in the future.

### 3.7. Adverse Effects

Adverse reactions associated with BoNT injections are rare, minimal, localized, transient, and dose-dependent. Ondo et al. [10] reported changes in the smiles of two patients. Shim et al. [7] reported three participants with masticatory difficulties. In a retrospective study of masseter hypertrophy, the main complications included perceived muscular weakness and aching, which were observed in 30% of the patients [33]. The second most common complication was bruising due to needle puncture of the vessels in the soft tissue, which occurred in 2.5% of the injections. Other complications, which occurred in less than 1% of the cases, included headache, asymmetrical smile, limited mouth opening, and xerostomia [33]. Recent studies have reported an association between mandibular bone loss and BoNT use in the masticatory muscles [34,35]. However, changes in the bone after BoNT injection are not considered adverse effects but a normal physiological response related to the excessive occlusal force correction [19]. BoNT injection reduces tension in the jaw elevator muscles and allows hypertrophied bones to revert to their original shape [23]. Asymmetrical smiles may be related to the diffusion of BoNT into the risorius or zygomatic muscles. Xerostomia can occur due to BoNT diffusion into the parotid gland [36]. Special attention should be paid during injection into the masseter muscle and during extraoral injection into the lateral pterygoid muscle [36]. Most of these side effects were probably due to the administration of a higher dosage of BoNT than necessary, the spread of BoNT from the target muscles into the bloodstream, or improper injection techniques. The clinician who administered the injections in this study was an expert who has administered BoNT to thousands of patients with bruxism or temporomandibular disorders; therefore, no such side effects were observed. Thorough knowledge of the local anatomy and injection techniques is crucial for minimizing side effects.

### 3.8. Future Directions

The heterogeneity across randomized controlled trials of BoNT therapy for bruxism regarding study design, BoNT dosages, injection sites and methods, follow-up duration, and the predominance of female participants makes drawing uniform conclusions and formulating standardized treatment protocols challenging [37]. All the analyzed studies used questionnaires, such as the VAS, to evaluate the effects of BoNT. However, these questionnaires are subjective and open to bias. In most previous studies, the sample sizes were relatively small. Therefore, clinical trials comparing different brands are necessary. All the studies demonstrated a positive effect of BoNT on primary bruxism, suggesting that BoNT injections can be considered an effective alternative modality for bruxism management. Standardized guidelines are warranted to maximize efficacy and minimize adverse effects [37].

Studies with compelling evidence that meet the following requirements are warranted to assess the general clinical application of BoNT therapy for bruxism [19]: (1) a definitive diagnosis by polysomnography with video recording or using ambulatory EMG devices; (2) exclusion of other pathological conditions, such as oromandibular dystonia or other neurological diseases; (3) placebo-controlled double-blind comparative studies with a statistically sufficient number of participants; (4) injection of BoNT by an experienced clinician; (5) optimal dosing; (6) sufficient research period and follow-up visits; (7) comprehensive evaluation of the changes in symptoms using a validated rating scale; and (8) difference in effect in some brands of BoNT.

## 4. Conclusions

BoNT therapy of the masseter muscles in patients with bruxism demonstrated a significant effect not only on muscle pain and discomfort but also on sleep quality, headaches, and shoulder and neck stiffness associated with bruxism. Factors influencing therapeutic effectiveness include age, sex, and muscle activity. More effective outcomes and side effects attributed to BoNT administration can be prevented by carefully examining patients before treatment and adjusting the optimal dosage for each patient.

## 5. Materials and Methods

### 5.1. Participants

A total of 304 patients with bruxism who visited the Furuhata Snoring and Sleep Disorder Research Institute and Furuhata Dental Clinic from October 2022 to December 2024 (206 women, 98 men, average age: 52.5 ± 15.9 years) were included in the study. Patients were examined and interviewed during their first visit. Bruxism was diagnosed based on the following inclusion and exclusion criteria: Inclusion criteria: (1) masticatory muscle pain or fatigue upon waking or during the day, (2) occlusal wear of two or more molars, (3) wear or breakage of the oral appliance in the case of users, (4) tenderness in the masseter and temporalis muscles on palpation testing, (5) tooth or root fracture, (6) bone protuberance, (7) wedge defect, (8) hypersensitivity in two or more sites, (9) definitive diagnosis of sleep bruxism by polysomnography, (10) family or partner reports of nighttime teeth grinding, and (11) those who have received conservative treatment such as splints or physical therapy in the past but have not achieved remission. The diagnosis of bruxism in this study was established through a comprehensive assessment integrating both subjective and objective criteria. In accordance with the international consensus [1], “probable” bruxism was defined as the presence of positive clinical signs (e.g., occlusal wear, and muscle tenderness) in combination with self-reported or bed-partner-reported symptoms. “Definite” sleep bruxism was diagnosed when instrumental verification, such as polysomnography with audiovisual recordings, was available. For the purposes of this study, patients were classified as having either “probable” or “definite” bruxism based on clinical examination, structured interviews, symptom reports from patients or their partners, and polysomnographic data when accessible. Exclusion criteria were as follows: (1) those with neuromuscular and psychiatric diseases and severe sleep disorders, (2) pregnant or breastfeeding women, (3) those who received BoNT-A injections into the masticatory muscles within the past 6 months, and (4) those under 18 years of age. To ensure diagnostic accuracy and exclude potential confounding conditions, one of the authors (K.Y.), a specialist in involuntary movements of the stomatognathic system, supervised the differential diagnostic process.

The patients received an explanation of the treatment plan and provided written informed consent. This study was performed in accordance with the Declaration of Helsinki after obtaining approval from the Institutional Review Board and Ethics Committee of Nippon Dental University (NDU-T2017-18).

### 5.2. Injection Method

Three injection sites were used on each side (one site at the maximal elevation of the masseter muscle and two sites at each apex of the base of a triangle with maximal elevation as the apex; the distance from the maximal elevation to the two bases was 1 cm); 100 units of BoNT-A (Botulax^®^, Hugel Inc., Seoul, Republic of Korea) was diluted with 2.5 mL of saline, and 0.6 mL was injected on each side (0.3 mL at the maximal elevation, 0.15 mL at the other two sites) using a 1 mL syringe attached with a 31-G, 12 mm needle.

### 5.3. Clinical Symptoms Measurement Items

The following items were measured before and 2 months after BoNT therapy.

#### 5.3.1. Maximal Action Potential of the Masseter Muscles

Specified surface electrode patches were attached to the left and right masseter muscle equivalents, and an electromyograph (Myonyx, MP JAPAN, Tokyo, Japan) (Figure 11A) was used to measure the maximal action potential by repeating a set of 5 s of clenching and 5 s of rest twice for 20 s (Figure 11A,B). The patch was attached in a uniform position for all patients such that the tragus, patch protrusion, and earth electrode were aligned in a straight line (Figure 11B) because BoNT-A administration may make palpation of the masseter muscle challenging. The average amplitude on both sides was calculated as the mean EMG amplitude for each patient. The skin surface was disinfected with alcohol and allowed to dry prior to the patch application. Measurements were obtained in a seated position with the head supported by a headrest, which the patients who used removable prostheses wore.

#### 5.3.2. Maximal Bite Force (N)

**Figure 12:**
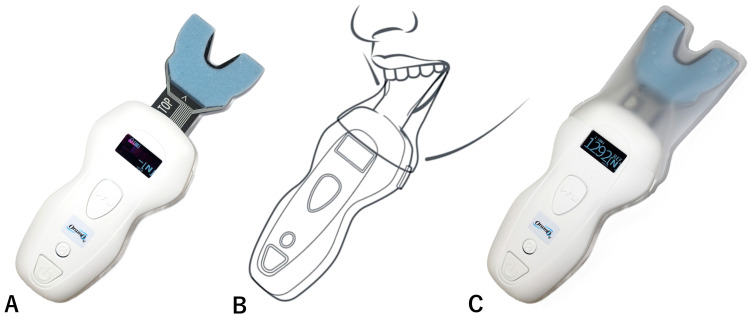
The bite force meter (Oramo-bf) (**A**) is attached with a sensor cover and inserted into the participant’s mouth to measure the maximal bite force (**B**); the result is then displayed (**C**).

The participants were asked to bite down on the sheet, and the maximal bite force was measured using a bite force meter (Oramo-bf: Yoshida, Tokyo, Japan) (Figure 12). Measurements were taken while the participants were in a seated position with their heads supported by a headrest.

#### 5.3.3. Tenderness of the Masticatory Muscles

**Figure 13:**
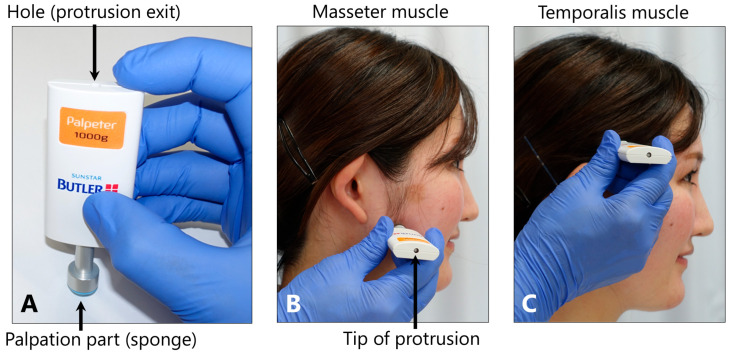
Evaluation of the tenderness in the masseter and temporalis muscles. The BUTLER Palpeter (**A**) presses the sponge-covered palpation part against the test muscle, and when the load reaches 1000 g, the tip of the protrusion appears from the hole (**B**). The presence of tenderness in the masseter muscle (**B**) and temporalis muscle (**C**) was ascertained.

The presence or absence of tenderness in the masseter and temporalis muscles was evaluated through interviews, using a skin algometer (BUTLER Palpeter 1000 g; Sunstar, Osaka, Japan) (Figure 13). Tenderness was confirmed using the 1000 g force applied to the origin, middle, and insertion of the masseter muscle and the anterior, middle, and posterior regions of the muscle belly of the temporalis muscle for 5 s each.

#### 5.3.4. Maximal Mouth Opening

The participants were asked to open their mouths as wide as possible, and the distance between the incisal edges of the upper and lower right central incisors was measured using a vernier caliper(Yamaura Instruments Co., Ltd., Tokyo, Japan). Measurements were taken with the participants in a seated position, with their heads supported by a headrest.

#### 5.3.5. Sleep Quality

Feeling refreshed upon awakening and how well they woke up were evaluated using the Visual Analogue Scale: VAS (10-point scale, with 10 being the best condition).

#### 5.3.6. Headache and Shoulder and Neck Stiffness

The patients were asked about the frequency and area of pain, and the pain intensity was evaluated using the VAS (10-point scale, with 10 indicating the strongest pain) (total of six blocks).

### 5.4. Statistical Analysis

- Changes in the Measurement Items after Treatment

The outcomes of the masseter muscle EMG amplitude, maximal bite force, maximal mouth opening, sleep quality, shoulder and neck stiffness, and headache were statistically analyzed using the Wilcoxon signed-rank sum test.

- Factors Affecting the Symptoms and Effects

Correlations among age, EMG amplitude before and after treatment, rate of decrease in the EMG amplitude, maximal bite force before and after treatment, rate of decrease in the maximal bite force, maximal mouth opening before and after treatment, rate of change in the maximal mouth opening, sleep quality before and after treatment, rate of change in sleep quality, shoulder and neck stiffness before and after treatment, rate of decrease in the stiffness, and headache before and after treatment.

Differences in age, EMG amplitude before and after treatment, rate of decrease in the EMG amplitude, maximal bite force before and after treatment, rate of decrease in the maximal bite force, maximal mouth opening before and after treatment, rate of change in the maximal mouth opening, sleep quality before and after treatment, rate of change in sleep quality, shoulder and neck stiffness before and after treatment, rate of decrease in the stiffness, and headache before and after treatment were analyzed using the Mann–Whitney U test.

All statistical analyses were performed using the statistical software package SPSS for Windows (version 24.0; SPSS Japan Inc., Tokyo, Japan) and Sigma Plot 12.0 (Systat Inc., Point Richmond, CA, USA).

## Figures and Tables

**Table 1:** Changes in the main measurement items after BoNT therapy compared to baseline.

	Baseline Mean (SD)	After BoNT Therapy Mean (SD)	*p*-Value
Mean EMG amplitude (μV)	189.0 (120.5)	55.4 (32.9)	*p* < 0.001
Maximal bite force (N)	618.4 (128.0)	527.3 (163.0)	*p* < 0.001
Maximal mouth opening (mm)	44.0 (7.8)	47.3 (7.6)	*p* < 0.001
Sleep quality (VAS: 0–10)	5.3 (2.0)	6.6 (1.8)	*p* < 0.001
Shoulder and neck stiffness (VAS: 0–10)	6.7 (2.9)	4.7 (2.9)	*p* < 0.001
Headache (VAS: 0–10)	5.4 (2.3)	2.9 (2.5)	*p* < 0.001

BoNT: botulinum neurotoxin, SD: standard deviation, EMG: electromyographic, VAS: visual analog scale.

**Table 2:** Results of the multivariate analysis affecting efficacy of botulinum neurotoxin (BoNT) therapy for bruxism.

	Estimate	Standard Error	Standardized Partial Regression Coefficients	*p*-Value
**Mean EMG amplitude reduction rate** (%)				
Constant	41.322	5.541		
Age (years)	0.269	0.0833	0.215	*p* = 0.001
Sex	−9.999	2.732	−0.23	*p* < 0.001
Mean EMG amplitude at baseline (µV)	0.0718	0.0119	0.396	*p* < 0.001
**Maximal bite force reduction rate** (%)				
Constant	−28.112	11.406		
Age (years)	0.438	0.11	0.28	*p* < 0.001
Sex	−9.845	3.618	−0.181	*p* = 0.007
Maximal bite force at baseline (N)	0.0356	0.0133	0.187	*p* = 0.008

EMG: electromyographic.

## Data Availability

The original contributions presented in this study are included in the article, and further inquiries can be directed to the corresponding author.

## Abbreviations

The following abbreviations are used in this manuscript:

BoNT	Botulinum neurotoxin
TMJ	Temporomandibular joint
EMG	Electromyography
